# Impaired mitochondrial integrity and compromised energy production underscore the mechanism underlying CoASY protein-associated neurodegeneration

**DOI:** 10.1007/s00018-025-05576-1

**Published:** 2025-02-22

**Authors:** Yuzhuo Shao, Jiaxin Hu, Kunhao Yan, Keke Zheng, Wenchi Sha, Jinlong Wang, Jiarui Wu, Yunpeng Huang

**Affiliations:** 1 Key Laboratory of Systems Health Science of Zhejiang Province, School of Life Science, Hangzhou Institute for Advanced Studies, University of Chinese Academy of Sciences, Hangzhou, 310024 China; 2 Key Laboratory of Systems Biology, Hangzhou Institute for Advanced Studies, University of Chinese Academy of Sciences, Chinese Academy of Sciences, Hangzhou, 310024 China

**Keywords:** Coenzyme A, CoASY, *Drosophila*, Mitochondria, ATP

## Abstract

Coenzyme A (CoA) is a crucial metabolite involved in various biological processes, encompassing lipid metabolism, regulation of mitochondrial function, and membrane modeling. CoA deficiency is associated with severe human diseases, such as Pantothenate Kinase-Associated Neurodegeneration (PKAN) and CoASY protein-associated neurodegeneration (CoPAN), which are linked to genetic mutations in Pantothenate Kinase 2 (PANK2) and CoA Synthase (CoASY). Although the association between CoA deficiency and mitochondrial dysfunction has been established, the underlying molecular alterations and mechanisms remain largely elusive. In this study, we investigated the detailed changes resulting from the functional decline of CoASY using the *Drosophila* model. Our findings revealed that a reduction of CoASY in muscle and brain led to degenerative phenotypes and apoptosis, accompanied by impaired mitochondrial integrity. The release of mitochondrial DNA was notably augmented, while the assembly and activity of mitochondrial electron transport chain (ETC) complexes, particularly complex I and III, were diminished. Consequently, this resulted in decreased ATP generation, rendering the fly more susceptible to energy insufficiency. Our findings suggest that compromised mitochondrial integrity and energy supply play a crucial role in the pathogenesis associated with CoA deficiency, thereby implying that enhancing mitochondrial integrity can be considered a potential therapeutic strategy in future interventions.

**Supplementary Information:**

The online version contains supplementary material available at 10.1007/s00018-025-05576-1.

## Introduction

Coenzyme A (CoA) is an essential and widely distributed cofactor that plays a pivotal role in biochemical processes, serving as an activator of molecules with carbonyl groups and as a carrier of acyl moieties, and participating in approximately 4% of cellular processes [1–3]. In particular, CoA forms high-energy thioester bonds with diverse carbonyl compounds that are involved in tricarboxylic acid cycle and oxidation, fatty acids and ketone bodies oxidation, acetyl-CoA synthesis, protein CoAlation, as well as the biosynthesis of fatty acids [4–7]. CoA deficiency is associated with a wide range of human diseases, encompassing neurodegeneration with brain iron accumulation (NBIA), acute myeloid leukemia (AML), and myopathy [7].

The synthesis of CoA is evolutionarily conserved, involving the sequential catalysis by enzymes including pantothenate kinase (PANK), phosphopantothenate cysteine synthase (PPCS), phosphopantothenate cysteine decarboxylase (PPCDC), phosphopantothenate adenyltransferase (PPAT), and dephosphorylated CoA kinase (Dpck) to stepwisely convert pantothenate (vitamin B5) to 4’-phosphopantothenate (PPan), and PPan is condensed with cysteine, decarboxylated to form PPanSH, and converted to dephospho-CoA and subsequently phosphorylated to form CoA-SH [7, 8]. Pantothenate is mainly uptaken from food by the intestine, thus highlighting the crucial role of external uptake of pantothenate for CoA synthesis [9–11].

Mutations in enzymes involved in CoA synthesis account for over 50% of NBIA cases, including pantothenate kinase 2 (PANK2), the enzyme responsible for the initial step, and CoA synthase (CoASY), the enzyme involved in the final step. These mutations are associated with two neurodegenerative disorders: pantothenate kinase-associated neurodegeneration (PKAN) and CoPAN [12, 13]. PKAN and CoPAN exhibit a spectrum of neurological symptoms, encompassing dystonia, Parkinsonism, dysarthria, behavioral disturbances, and motor axonal neuropathy; however, the underlying molecular mechanism remains elusive [14]. Excessive iron deposition in the brain, particularly within the globus pallidus, is also a common feature observed in PKAN and CoPAN, however, it may occur as a secondary phenomenon rather than being an initial symptom [14].

Additionally, the pathogenesis associated with CoA deficiency encompasses various cellular alterations, such as mitochondrial dysfunction, heightened oxidative stress, altered iron metabolism, and impaired fatty acid and lipid metabolism [7, 15]. The mitochondrion, as the central organelle, plays a pivotal role in the synthesis of CoA and ATP, serving as the primary energy source for most of the biological processes [16, 17]. Although impaired mitochondrial function and mitochondrial fatty acid metabolism have been observed in various PKAN models [18], the underlying mechanism remains elusive. Crucially, it remains to be determined whether the functional decline of CoASY in accordance with the PANK2 mutation leads to mitochondrial dysfunction and if the deficiency in energy supply plays a critical role in PKAN and CoPAN pathogenesis.

In addition to brain atrophy, patients with CoASY mutation also exhibit muscular disorders. However, the underlying mechanism remains to be elucidated [19]. Furthermore, despite the documented significant perturbation of mitochondrial function in PANK2 animals, it remains unclear whether CoASY can impair mitochondrial function and integrity. Moreover, the underlying mechanism behind this potential impairment is also uncertain. In addition, the relationship between reduced mitochondrial function and muscle disorders resulting from CoASY deficiency remains to be elucidated. Interestingly, the human CoASY gene encodes a bifunctional enzyme that encompasses both PPAT and Dpck activities, distinguishing it from the bacterial CoASY, which consists of two separate enzymes responsible for catalyzing the final two steps of de novo CoA biosynthesis [20]. Similar to humans, CoASY also exhibits bifunctional enzyme activity and is encoded by a single CoASY gene named Dpck in *Drosophila melanogaster* [21], a well-established model organism that facilitates the observation of corresponding phenotypes and investigation of molecular alterations. Thereby, to further elucidate the feature and underlying mechanism of CoASY-induced functional decline, we generated a *Drosophila* model with muscle and central nervous system (CNS)-specific knockdown of CoASY. Our findings demonstrate the crucial involvement of mitochondrial ETC integrity deterioration and impaired ATP production in the context of reduced CoASY levels.

## Results

### Phenotypic characterization of ubiquitous Dpck knockdown

To investigate the biological function of CoASY in the *Drosophila* model, we initially conducted a blast analysis on Flybase to identify the homologue of human CoASY. Our analysis revealed that CG1939 is the only conserved homolog in the fly genome, and its homolog is present across a wide range of organisms, from Archaea to humans (Supplementary Fig. 1A and 1B). According to the Flybase nomenclature, we employed Dpck as a representative of fly CoASY in subsequent studies. For further functional analysis, two Dpck RNAi lines were collected, and the overexpression transgene was generated using the pUAST plasmid.

**Fig. 1:**
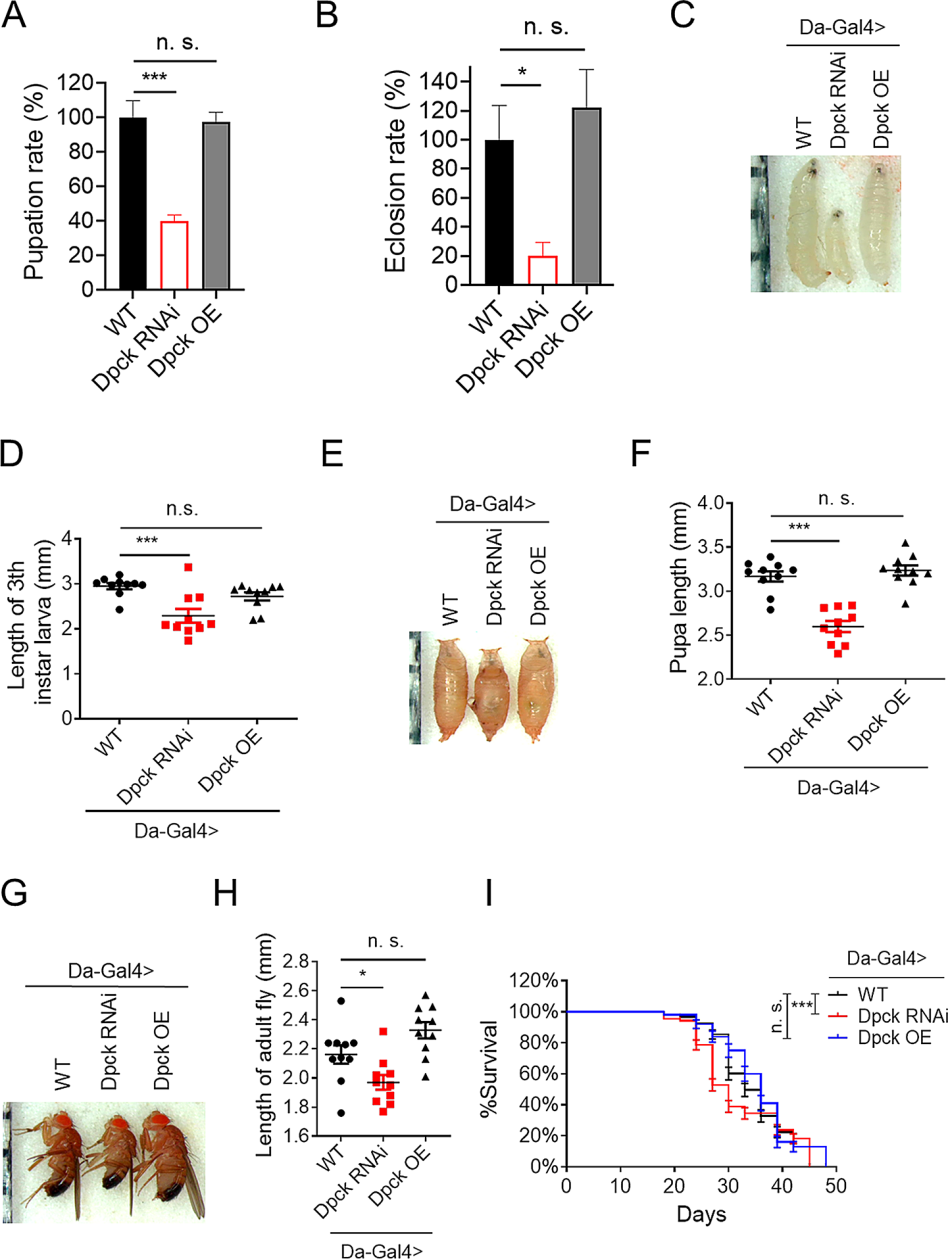
Phenotypic characterization of ubiquitous Dpck knockdown (**A**). Pupation rate of Dpck RNAi flies. ***: *p* < 0.001. *n* = 300 (6 biological replicates). (**B**). Eclosion rate of Dpck RNAi flies. *: *p* < 0.05. *n* = 300 (6 biological replicates). (**C**-**H**). Dpck RNAi reduces fly body size. *n* = 10. (**C**, **D**). The length of the 3rd instar larva is measured. (**D**) is the quantitative result of (**C**). ***: *p* < 0.001. (**E**, **F**). The length of the fly pupa is measured. (**F**) is the quantitative result of (**E**). ***: *p* < 0.001. (**G**). The length of adult flies is measured. (**H**) is the quantitative result of (**G**). *: *p* < 0.05. *n* = 10. (**I**). Lifespan of Dpck RNAi flies. *n* = 100 (5 biological replicates). The Gehan-Breslow-Wilcoxon test is employed for the analysis of differences in lifespan. ***: *p* < 0.001. Da-Gal4 is used to drive the ubiquitous overexpression and knockdown of Dpck presented in Fig. 1

The RT-PCR analysis revealed a significantly high RNAi efficiency in RNAi line 2 (Supplementary Fig. 1C). Consistently, the impairment of fly climbing ability was more severe when using RNAi line 2, resulting in a reduction of climbing ability from approximately 90–12% (Supplementary Fig. 1D). Therefore, RNAi line 2 was used in subsequent investigations. Repression of Dpck not only impairs fly climbing ability but also hampers the developmental process. The pupation rate and eclosion rate were both significantly reduced following Dpck RNAi (Fig. 1A and B). Serving as the control, the eclosion rate and pupation rate were not impacted by GFP RNAi (Supplementary Fig. 1E and 1 F). Furthermore, knockdown of Dpck resulted in a reduction in the body size of 3rd instar larvae, pupae, and adult flies (Fig. 1C-H), indicating that down-regulation of Dpck hampers normal developmental processes. The climbing ability was also impaired upon Dpck knockdown (Supplementary Fig. 1G), while GFP RNAi had no impact on the locomotor performance of flies. The longevity of flies was also influenced by Dpck RNAi. Lifespan measurements revealed a significant reduction in the median lifespan of flies following Dpck RNAi, with a decrease from approximately 34 days to around 30 days (Fig. 1I). This observation, coupled with the decline in fly climbing ability, suggests that diminished levels of Dpck can lead to functional degeneration.

The impact of Dpck overexpression was also assessed. Ubiquitous overexpression of Dpck did not significantly affect fly development, as evidenced by the unaffected pupation rate, eclosion rate, and length of 3rd instar larvae and adult flies (Fig. 1A-H). Interestingly, ubiquitous Dpck overexpression resulted in impaired fly climbing ability (Supplementary Fig. 1C and 1D).

### Repression of Dpck results in the attenuation of muscle function

**Fig. 2:**
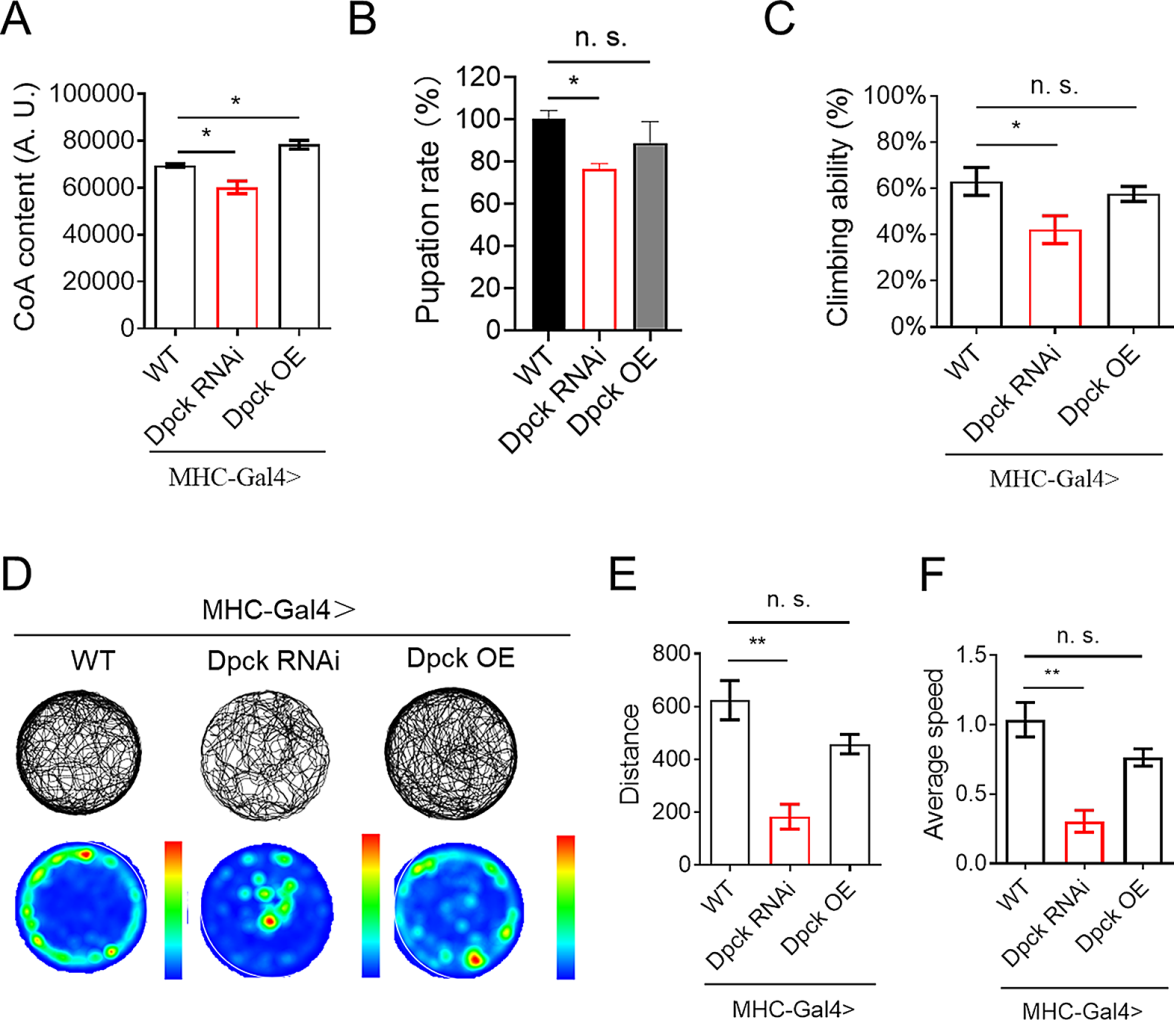
Repression of Dpck leads to the attenuation of muscle function. (**A**). CoA content in the thoraxes of the flies with Dpck RNAi and OE. *: *p* < 0.05. *n* = 20 (3 biological replicates). (**B**). The pupation rate of flies with muscle-specific knockdown of Dpck. *: *p* < 0.05. *n* = 300 (6 biological replicates). (**C**) Climbing ability of flies with muscle-specific Dpck RNAi. *: *p* < 0.05. *n* = 100 (5 biological replicates). (**D**). Open-field analysis of flies with muscle-specific Dpck RNAi. *n* = 5. (**E**) moved distance and (**F**) average speed are the quantitative results of (**D**). **: *p* < 0.01. MHC-Gal4 is used to drive the overexpression and knockdown of Dpck in the fly’s indirect flight muscles

Given the critical role of CoA in maintaining mitochondrial function, it has been proposed that a deficiency in CoA may impair mitochondrial function, particularly when there is a high demand for functional mitochondria in muscle [22], we conducted further investigations to explore the impact of muscle-specific knockdown of Dpck using MHC-Gal4 (Supplementary Fig. 2A). The CoA content in muscle tissues was significantly reduced by Dpck RNAi and increased by Dpck overexpression (Fig. 2A). Interestingly, knockdown of Dpck in fly indirect muscle recapitulated several phenotypes observed in ubiquitous Dpck knockdown, including the impairment of fly developmental processes, resulting in a reduction of pupation rate from approximately 100% to around 77% (Fig. 2B). Furthermore, knockdown of Dpck in fly muscle tissue resulted in a significant decline in climbing ability, with the climbing index decreasing from 60 to 40% (Fig. 2C). The pupation rate and climbing ability were not affected by GFP RNAi in muscle tissue, which was served as the control (Supplementary Fig. 2B and 2C). Conversely, overexpression of Dpck did not exert any influence on climbing ability (Fig. 2C). The open field test revealed that Dpck RNAi disrupted fly behavior, as evidenced by altered trajectory (Fig. 2D-F), reduced moving distance and average speed (Fig. 2E and F). These findings suggest that Dpck RNAi can impair the functional integrity of muscle. Interestingly, overexpression of Dpck in fly muscle did not result in significant changes to development, climbing ability, and behavior, despite an increase in CoA concentration within the muscle (Fig. 2A-F).

### Repression of Dpck disrupts mitochondrial integrity and impairs its functionality

**Fig. 3:**
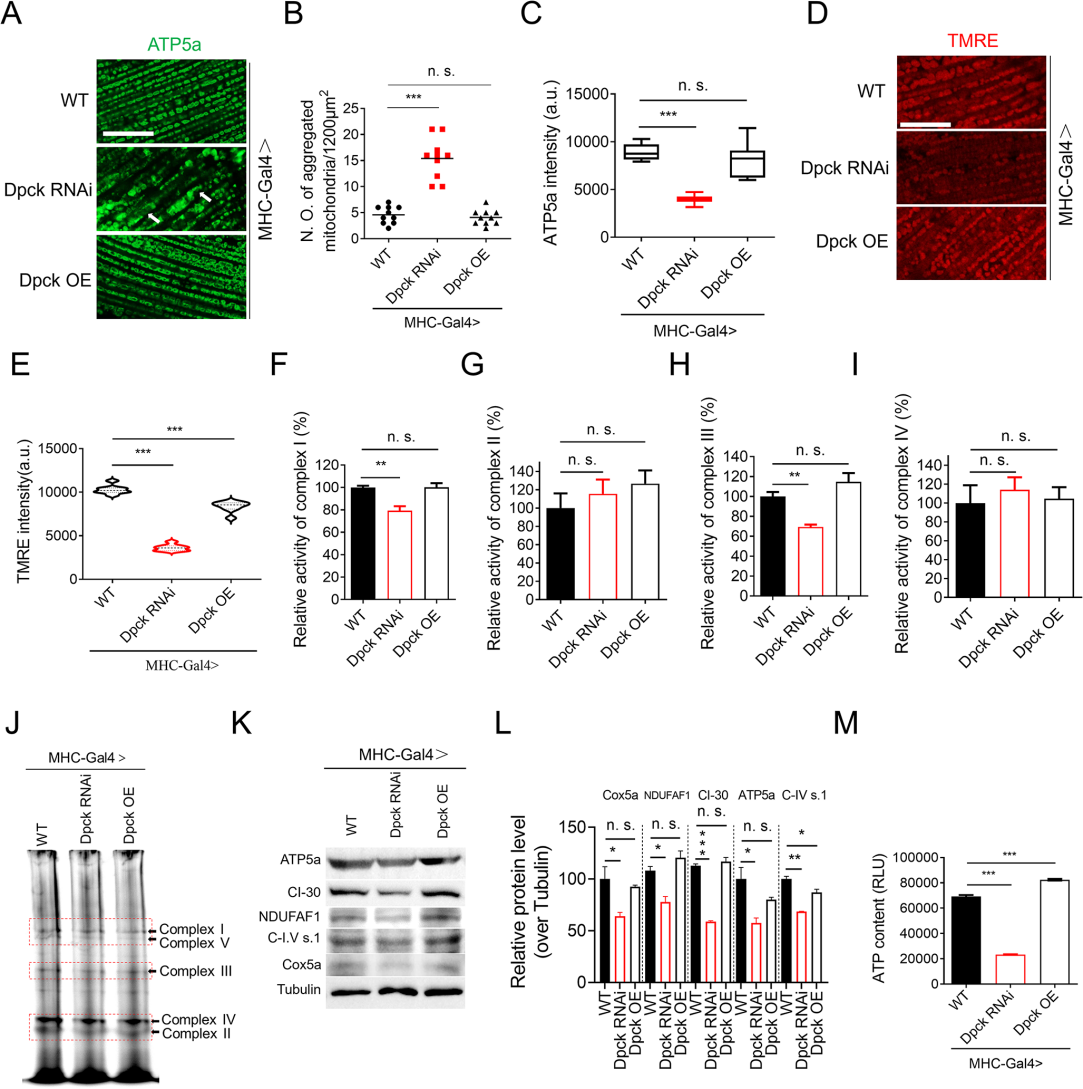
Repression of Dpck disrupts mitochondrial integrity and impairs its functionality. (**A**). Mitochondrial morphology was visualized using ATP5a immunofluorescence, scale bar = 15 μm. *n* = 10. (**B**, **C**). The quantitative results of (**A**). (**B**) is the number of aggregated mitochondria. ***: *p* < 0.001. (**D**). Muscle mitochondrial membrane potential was assessed through TMRE staining, scale bar = 15 μm. *n* = 8. (**E**) is the quantitative result of (**D**). ***: *p* < 0.001. (**F**-**I**). The activities of muscle mitochondrial electron transport chain complex I-IV were analyzed. **: *p* < 0.01. MHC-Gal4 was utilized to drive both overexpression and knockdown of Dpck in fly indirect flight muscle. *n* = 50 (3 biological replicates). (**J**). The integrity of muscle mitochondrial electron transport chain complexes was evaluated using BN-gel electrophoresis, followed by Coomassie Brilliant Blue staining. Complexes I-V are indicated by arrows. *n* = 120 (3 biological replicates). (**K**). Protein levels of ETC subunits were examined via western blotting. *n* = 60 (3 biological replicates). (**L**) is the quantitative result of (**K**). *: *p* < 0.05, **: *p* < 0.01, ***: *p* < 0.001. (**M**). Measurement of ATP content in the thoraxes of flies with Dpck RNAi and overexpression. ***: *p* < 0.001. *n* = 20 (3 biological replicates)

Considering the observed impairment in muscle function upon Dpck knockdown, we subsequently conducted a comprehensive investigation into the integrity and functionality of mitochondria in Dpck RNAi muscle tissues. Interestingly, Dpck RNAi led to significant mitochondrial aggregation and a decrease in ATP5a staining intensity (Fig. 3A-C). However, the overexpression of Dpck did not induce any changes in mitochondrial morphology or ATP5a intensity (Fig. 3A-C). Given that mitochondrial aggregation is indicative of disrupted mitochondrial function [23], we proceeded to further investigate the activity of the mitochondrial electron transport chain (ETC). Firstly, the mitochondrial membrane potential was assessed using tetramethylrhodamine ethyl ester perchlorate (TMRE) staining. Dpck RNAi significantly attenuated the TMRE signal, indicating a decline in mitochondrial membrane potential (Fig. 3D and E). Surprisingly, Dpck overexpression also partially diminished the membrane potential to a certain extent (Fig. 3D and E).

Additionally, the activity of complex I and III exhibited a reduction in Dpck RNAi muscle tissues, while no significant alterations were observed in the activity of complex II and IV (Fig. 3F and I). Unexpectedly, the overexpression of Dpck did not increase the activity of ETC complexes (Fig. 3F-I). Consistently, further analysis using Blue Native PAGE (BN-PAGE) revealed that knockdown of Dpck resulted in compromised integrity of ETC complexes, particularly complex I and complex III (Fig. 3J). Moreover, the expression levels of ETC proteins were also diminished upon Dpck RNAi (Fig. 3K and L), including CI-30, NDUFA1, C-IV s. 1, Cox5a, and ATP5a. Corresponding to the decline in mitochondrial function, depletion of Dpck significantly reduced ATP content (Fig. 3M). The findings presented here provide further evidence that Dpck RNAi can attenuate mitochondrial integrity and function, leading to impaired ATP production. Interestingly, while the overexpression of Dpck did not enhance the activity and integrity of ETC complexes (Fig. 3D-J), it significantly elevated ATP content (Fig. 3M), potentially due to an increase in CoA concentration and metabolic regulation. Given that ATP can also be generated through lipid and fatty acid metabolism, we conducted an analysis on the levels of lipid droplets (LDs) in fly muscle tissues. Immunostaining of LDs in fly muscle tissues indicated a reduction in LD abundance upon overexpression of Dpck (Supplementary Fig. 3A and 3B). Additionally, we conducted further analysis on the levels of triacylglycerol (TAG) and free fatty acid. Our findings demonstrated that Dpck-OE resulted in a reduction in TAG levels and an increase in free fatty acid levels (Supplementary Fig. 3C and 3D), concomitant with elevated NADH and Ac-CoA levels (Supplementary Fig. 3E-G), while maintaining comparable mitochondrial membrane potential (Supplementary Fig. 3H). Consequently, enhanced lipid metabolism may lead to an augmentation in ATP levels [24–26].

### Repression of Dpck leads to muscle degeneration

**Fig. 4:**
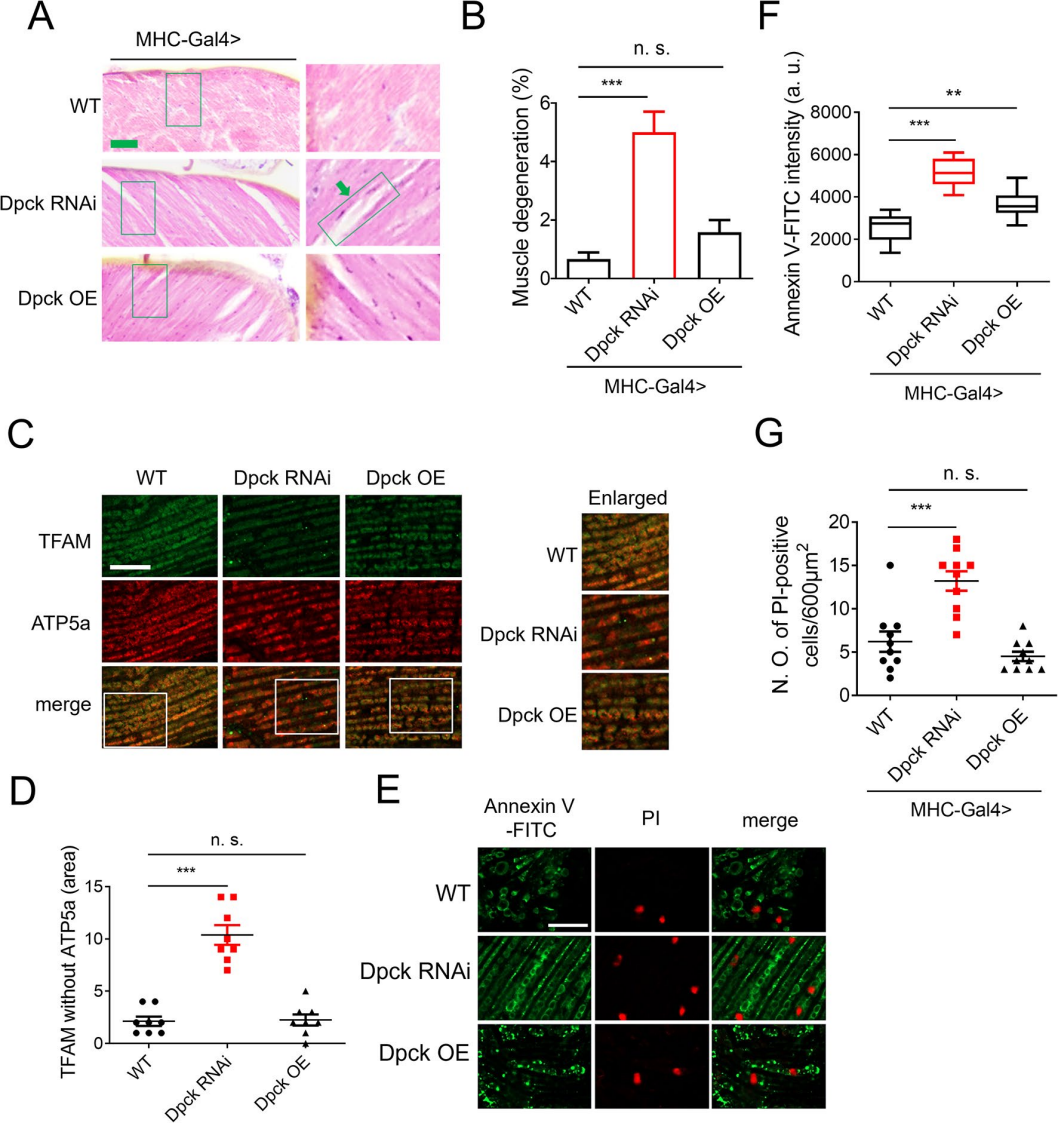
Repression of Dpck leads to muscle degeneration. (**A**). H&E staining demonstrates degenerative phenotypes in the thoracic section of Dpck RNAi and OE flies, indicated by arrows. Scale bar = 80 μm. *n* = 20. (**B**). Quantitative analysis of (**A**). ***: *P* < 0.001. (**C**). Mitochondrial DNA release is visualized through TFAM staining, with TFAM and ATP5a serving as markers for mitochondrial DNA and mitochondria, respectively. Scale bar = 20 μm. *n* = 8. (**D**). Quantitative analysis of the extra-mitochondrial TFAM signal in (**C**). ***: *p* < 0.001. MHC-Gal4 is utilized to drive Dpck overexpression and knockdown in the indirect flight muscle. (**E**). Apoptotic phenotypes in muscle tissue are assessed using Annexin V-FITC and PI staining. Scale bar = 15 μm. *n* = 10. (**F**, **G**). Quantitative results of (**F**). **: *p* < 0.01, ***: *p* < 0.001

Considering the significant decline in mitochondrial function upon Dpck RNAi, we hypothesized that this intervention may lead to muscle degeneration. To investigate this possibility, we performed hematoxylin and eosin (H&E) staining on muscle sections to assess the presence of degenerative phenotypes. Notably, Dpck RNAi induced pronounced muscle degeneration, whereas Dpck overexpression did not elicit such pathological changes (Fig. 4A and B).

Considering the disruption of mitochondrial integrity induced by Dpck RNAi, we subsequently assessed the proportion of released mitochondrial DNA (mtDNA) through TFAM staining. Notably, Dpck RNAi significantly elevated the levels of extra-mitochondrial TFAM, implying an augmentation in mtDNA release (Fig. 4C and D). Conversely, overexpression of Dpck did not elicit any discernible alterations. Further investigation revealed that Dpck RNAi increased the intensity of Annexin V-FITC and the proportion of PI-positive cells (Fig. 4E-G), indicating that reducing Dpck expression enhances apoptosis events in muscle tissues, consistent with muscle degeneration. Interestingly, Dpck overexpression also slightly elevated the intensity of Annexin V-FITC but not the proportion of PI-positive cells (Fig. 4E-G), which may be attributed to an increase in CoA concentration.

### Repression of Dpck induces neurodegeneration

**Fig. 5:**
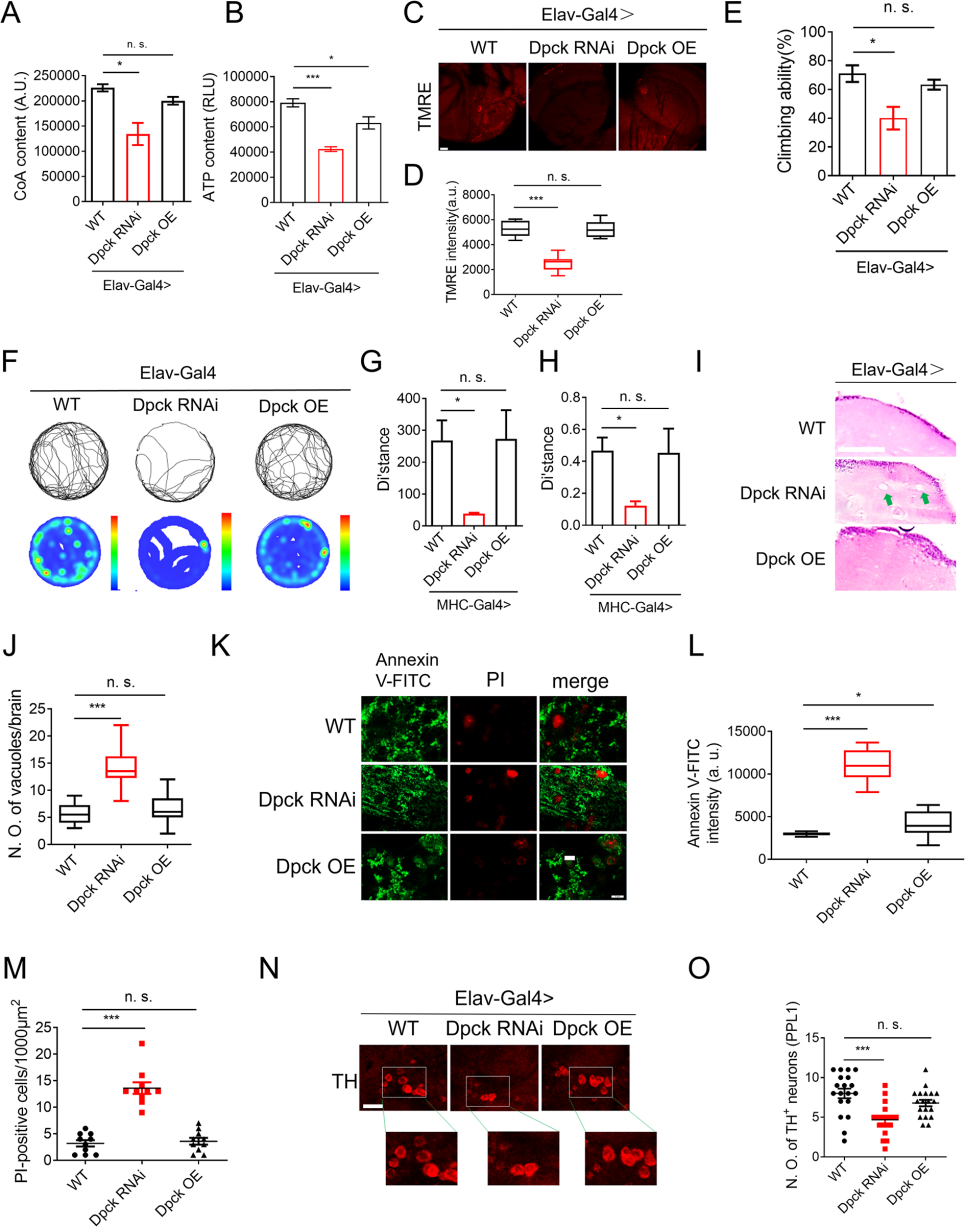
Repression of Dpck induces neurodegeneration. (**A**). CoA content in fly heads was measured after RNAi and overexpression (OE) of Dpck specifically in the central nervous system (CNS). *: *p* < 0.05. *n* = 20. (**B**). ATP content in fly heads was assessed following CNS-specific RNAi and OE of Dpck. *: *p* < 0.05, ***: *p* < 0.001. (**C**). TMRE staining was performed on fly brains, with a scale bar indicating 20 μm. *n* = 10. (**D**) presents the quantitative results obtained from (**C**), where statistical significance is indicated by *** for *p* < 0.001. (**E**). The climbing ability of flies was evaluated after CNS-specific RNAi and overexpression of Dpck, with statistical significance denoted by * for *p* < 0.05. *n* = 100 (5 biological replicates). (**F**). Open field assay was conducted to analyze the behavior of flies subjected to CNS-specific RNAi and overexpression of Dpck. *n* = 3 (3 biological replicates). (**G**) represents the distance traveled during open field assay, while (**H**) displays the average speed observed during this test. *: *p* < 0.05. (**I**). H&E staining was performed on brain sections to reveal degenerative phenotypes through vacuole quantification, with a scale bar indicating 30 μm. *n* = 20. (**J**) provides quantitative results derived from (**I**), where statistical significance is represented as *** for *p* < 0.001. (**K**). Apoptotic phenotypes in fly brains are detected using Annexin V-FITC and PI staining, with a scale bar indicating 5 μm. *n* = 10. (**L**, **M**) present the corresponding quantitative results obtained from (**K**), where statistical significance is indicated by * for *P* < 0.05 and *** for *P* < 0.001. (**N**). The number of posterior inferiorlateral protocerebrum (PPL1) neurons was analyzed via TH immunofluorescence imaging, with a scale bar indicating 10 μm. *n* = 20. (**O**) shows the quantitative result derived from (**N**), where statistical significance is represented as *** for *p* < 0.001

Given the association of the Dpck mutation with neurodegeneration, we conducted further investigations to ascertain whether Dpck RNAi could induce a degenerative phenotype in the brain. To achieve this, we employed the Elav-Gal4 driver to specifically downregulate and overexpress Dpck within the central nervous system (CNS) (Supplementary Fig. 4A). The knockdown of Dpck resulted in a significant decrease in the CoA level in fly heads (Fig. 5A), concomitant with a reduction in ATP content (Fig. 5B) and a decline in mitochondrial membrane potential (Fig. 5C and D).

Furthermore, knockdown of Dpck resulted in a significant decrease in climbing ability from 70 to 40% (Fig. 5E). As the control, GFP RNAi in the CNS system did not elicit a comparable defect (Supplementary Fig. 4B). Revealingly, the pupation rate was also notably reduced upon specific knockdown of Dpck in the CNS (Supplementary Fig. 4C). Moreover, the open field assay demonstrated disrupted fly behavior upon Dpck RNAi (Fig. 5F-H). Specifically, both the moved trajectory and distance were significantly disturbed, along with a decrease in average speed (Fig. 5G-H), indicating that decreased expression of Dpck within the CNS also leads to degeneration.

Specifically, H&E staining of brain sections revealed a significant increase in the number of degenerative vacuoles within Dpck RNAi (Fig. 5I and J), with vacuole numbers increasing from ~ 5 to ~ 13 per brain. In contrast, overexpression of Dpck did not significantly affect the rate of vacuolization (Fig. 5I and J), further supporting the notion that the functional decline of Dpck can lead to neurodegeneration. Moreover, the ratio of apoptotic cells was further augmented by Dpck RNAi treatment, as evidenced by the enhanced intensity of Annexin V-FITC and an increased number of PI positive cells (Fig. 5K-M). Notably, while Dpck overexpression marginally elevated the intensity of Annexin V-FITC, it did not result in an increase in the number of PI positive cells, consistent with observations made in muscle tissue (Fig. 5K-M).

Importantly, Dpck RNAi resulted in a reduction in the number of inferiorlateral protocerebrum (PPL1) neurons, as evidenced by tyrosine hydroxylase (TH) staining, thereby suggesting that Dpck RNAi can induce neuronal loss (Fig. 5N and O). Conversely, overexpression did not elicit any significant alteration. Our findings collectively indicate that the depletion of Dpck is associated with neurodegeneration. Notably, neuronal knockdown of Dpck also impairs developmental processes, as evidenced by a reduction in fly pupation rate (Supplementary Fig. 4C and 4D).

### Expression of human CoASY complements Dpck RNAi

**Fig. 6:**
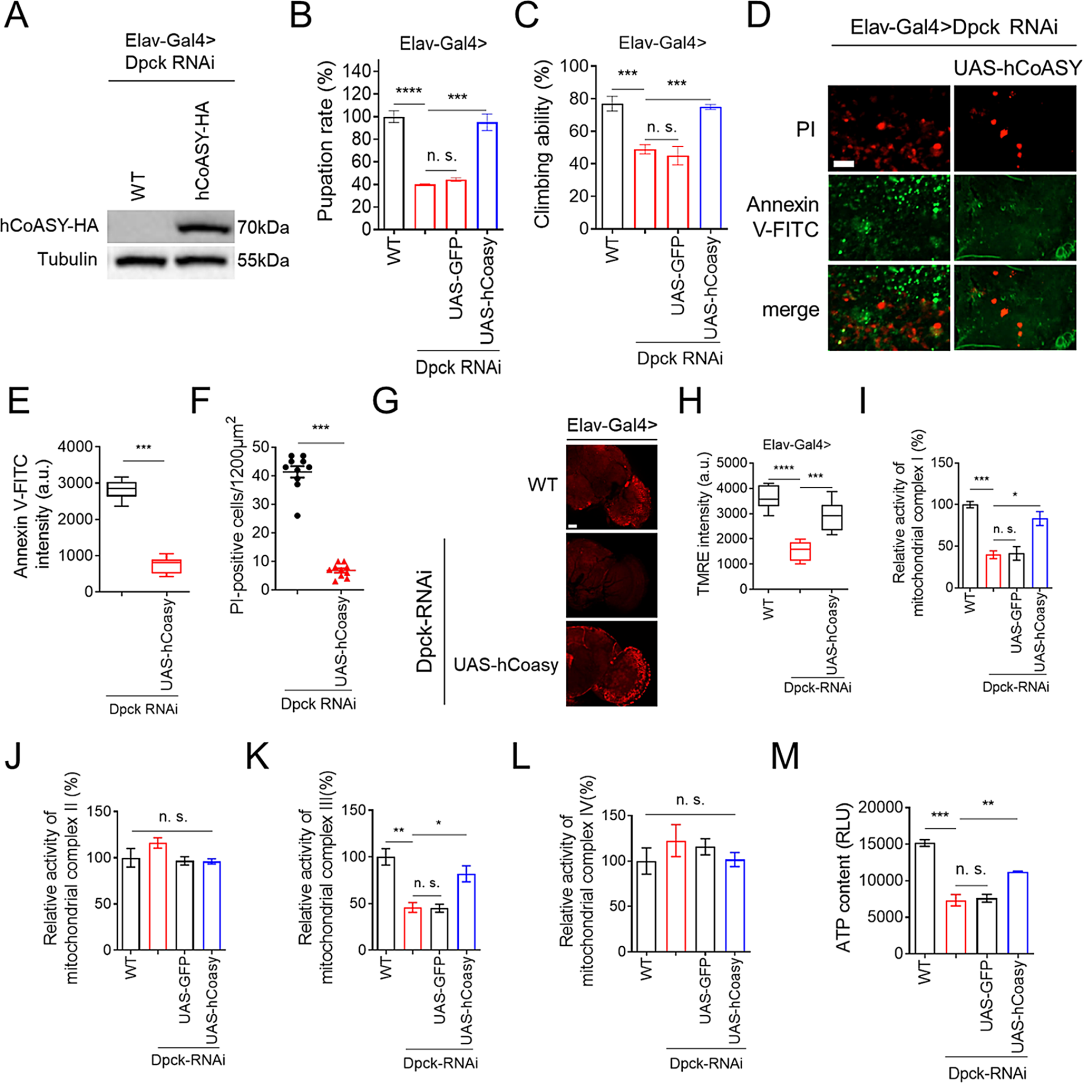
Human CoASY effectively rescues the defects induced by Dpck RNAi. (**A**). The expression of human CoASY (hCoASY) in transgenic flies is analyzed by immunoblotting, with tubulin serving as the loading control. Elav-Gal4 is employed to drive hCoASY expression specifically in the CNS of *Drosophila*. *n* = 20 (3 biological replicates). (**B**, **C**). The pupation rate and climbing ability of Dpck RNAi flies can be restored by hCoASY. Elav-Gal4 is used to induce gene expression and knockdown in the fly CNS. ***: *p* < 0.001; ****: *p* < 0.0001. *n* = 250 (5 biological replicates). (**D**-**F**). The apoptotic phenotype of Dpck RNAi flies can be ameliorated by hCoASY. Elav-Gal4 is used to induce gene expression and knockdown in the fly CNS. *n* = 8. (**D**). The PI and Annexin V-FITC staining of fly brain. Scale bar = 10 μm. (**E**). is the quantitative analysis of the intensity of Annexin V-FITC. ***: *p* < 0.001. (**F**). is the quantitative results of the number of PI positive cells. ***: *p* < 0.001. (**G**, **H**). The assessment of mitochondrial membrane potential in the fly brain is conducted using TMRE staining. Elav-Gal4 is employed to drive gene expression and knockdown in the fly CNS. Scale bar = 50 μm. *n* = 8. (**H**). is the quantitative result of (**G**). ***: *p* < 0.001; ****: p < 0.0001. (**I**-**L**). The activity of electron transport chain (ETC) complexes I-IV is analyzed. Elav-Gal4 is employed to drive gene expression and knockdown in the fly CNS. *n* = 50 (3 biological replicates). (**I**). complex I activity, (**J**). complex II activity, (**K**). complex III activity, (**L**). complex IV activity. *: *p* < 0.05; **: *p* < 0.01; ***: *p* < 0.001. (**M**). Quantification of ATP levels in the heads of Dpck RNAi flies and hCoASY-expressing flies. **: *p* < 0.01; ***: *p* < 0.001. *n* = 20 (3 biological replicates)

To assess the functional similarity between human CoASY (hCoASY) and fly Dpck, we subsequently generated UAS-hCoASY-HA transgenic flies and confirmed the expression of hCoASY through immunoblot analysis (Fig. 6A). The pupation rate and climbing ability of CNS-specific Dpck RNAi flies were successfully restored by hCoASY, with the relative pupation rate increasing from approximately 40% to around 95% (Fig. 6B), and the climbing ability improving from about 50–72% (Fig. 6C). These improvements were accompanied by a reduction in apoptosis observed in fly brains (Fig. 6D-F). Furthermore, we also investigated whether impaired mitochondrial function could be rescued by hCoASY. Interestingly, the expression of hCoASY in flies significantly restored mitochondrial membrane potential and the activity of mitochondrial ETC complex I and III (Fig. 6G-L). Consistently, ATP content was also elevated (Fig. 6M), suggesting that hCoASY expression can effectively complement the defect resulting from fly Dpck RNAi.

### The knockdown of Dpck enhances the susceptibility to starvation and oxidative stress

Considering the observed decline in mitochondrial function and ATP production upon Dpck RNAi, we postulated that the Dpck RNAi flies would exhibit heightened susceptibility to energy deprivation and oxidative stress.

**Fig. 7:**
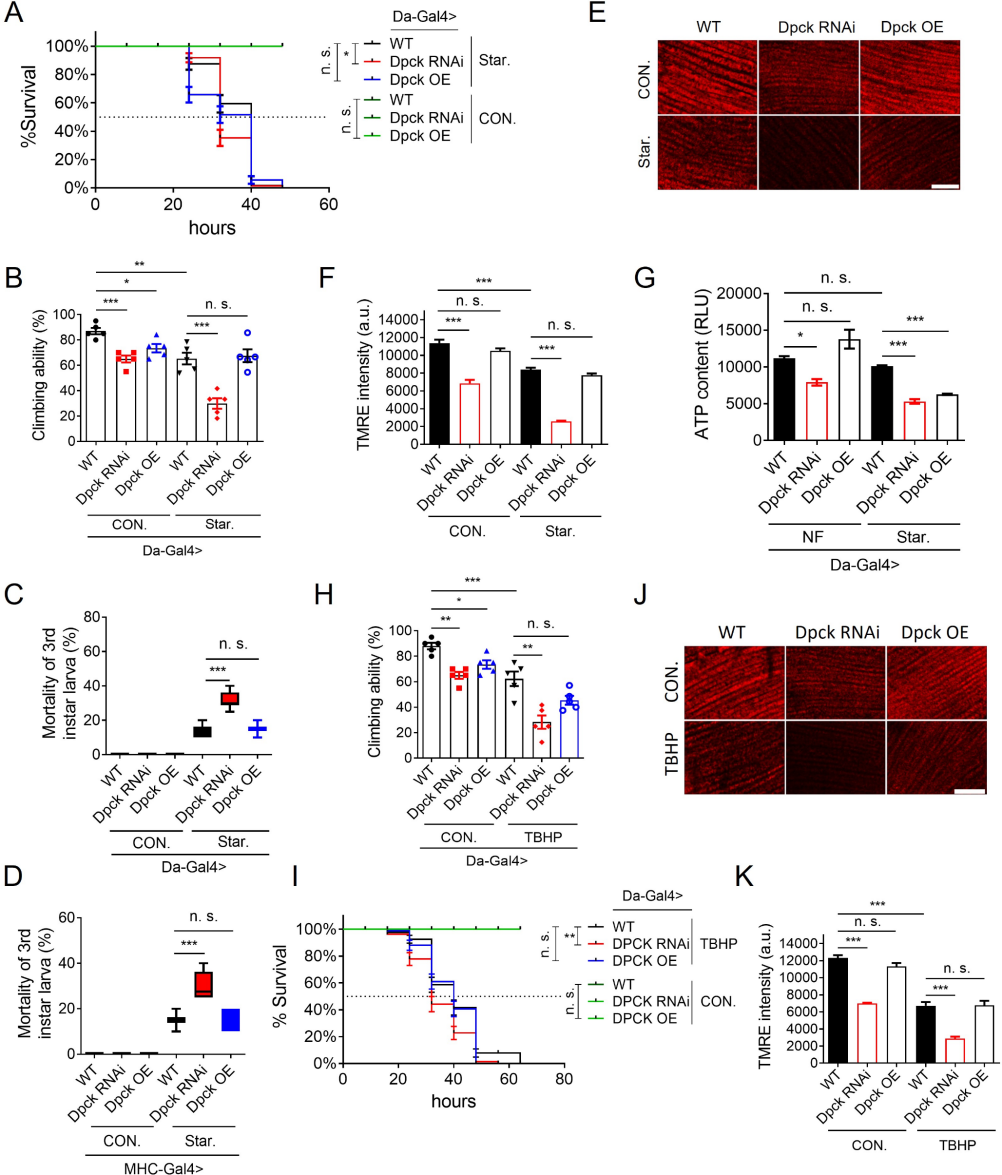
The knockdown of Dpck enhances the susceptibility to starvation and oxidative stress. **(A)**. Survival rate of Dpck RNAi flies under the stress of starvation. The Gehan-Breslow-Wilcoxon test is employed for the analysis of differences in lifespan. *: *p* < 0.05. *n* = 100 (5 biological replicates). **(B)**. The climbing ability of flies under starvation stress. *: *p* < 0.05, **: *p* < 0.01, ***: *p* < 0.001. *n* = 100 (5 biological replicates). **(C, D)**. is the mortality of 3rd instar larvae under starvation stress. ***: *p* < 0.001. *n* = 300 (6 biological replicates). **(C)**. Dpck is ubiquitously knocked down by using the Da-Gal4 driver. **(D)**. Dpck is specifically knocked down in the fly’s indirect flight muscles. **(E)**. Mitochondrial membrane potential is revealed by TMRE staining. Scale bar = 20 um. *n* = 10.** (F)**. is the quantitative result of **(D)**. ***: *p* < 0.001. **(G)**. ATP content in starved fly bodies is analyzed. *: *p* < 0.05, ***: *p* < 0.001. *n* = 15 (3 biological replicates). **(H)**. Climbing ability of flies treated by TBHP stress. *: *p* < 0.05, **: *p* < 0.01. *n* = 100 (5 biological replicates). **(I)**. Survival rate of flies treated with tert-butyl hydroperoxide (TBHP). The Gehan-Breslow-Wilcoxon test is employed for the analysis of differences in lifespan. **: *p* < 0.01. *n* = 100 (5 biological replicates). **(J)**. TBH-induced changes of mitochondrial membrane potential are revealed by TMRE staining. Scale bar = 20. um. *n* = 10. **(K)** is the quantitative result of** (J)**. ***: *p* < 0.001. Da-Gal4 is used to drive the RNAi and OE of genes

Therefore, Dpck was ubiquitously knocked down in flies using the Da-Gal4 driver, and subsequently subjected to a starvation treatment. The survival rate of Dpck RNAi flies was significantly reduced by starvation. The median survival time was reduced from ~ 40 h to ~ 32 h (Fig. 7A). Furthermore, the climbing ability of WT flies was significantly impaired by starvation, with a decrease from 86 to 64%. In addition, the decline in climbing ability was more pronounced upon knockdown of Dpck, resulting in a reduction from approximately 62% to around 28% (Fig. 7B). These findings strongly suggest that Dpck RNAi enhances sensitivity to starvation. Interestingly, the overexpression of Dpck was found to confer a higher resistance to starvation, with only a marginal reduction in climbing ability from 70 to 62% (Fig. 7B).

Furthermore, ubiquitous Dpck RNAi also resulted in a significant increase in the mortality rate of 3rd instar larvae under starvation conditions, which escalated from 12 to 30% (Fig. 7C). Knockdown of Dpck specifically in the indirect flight muscle also led to an elevated mortality rate (Fig. 7D), rising from 12 to 25%. In consistence, the starvation treatment resulted in a significant reduction in mitochondrial membrane potential and ATP content, indicating that the functional decline of Dpck is more susceptible to energy deprivation (Fig. 7E-G).

Interestingly, knockdown of Dpck also augmented the susceptibility to tert-butyl hydroperoxide (TBHP) treatment. The impact on fly climbing ability and survival rate was particularly pronounced upon down-regulation of Dpck (Fig. 7H and I), resulting in a reduction of the median survival rate from 40 h to 31 h (Fig. 7I). Furthermore, the climbing ability was reduced from 65 to 28%, whereas the climbing capacity of WT flies decreased from 88 to 65% (Fig. 7H). The treatment with TBHP significantly attenuated the mitochondrial membrane potential as well (Fig. 7J and K), indicating that the reduction of Dpck also enhances susceptibility to ROS-induced damage. Our findings demonstrate that Dpck plays a critical role in the regulation of mitochondrial integrity and energy production. Impaired functionality of Dpck disrupts mitochondrial function, leading to an energy deficit and subsequent degeneration, which serves as a critical mediator involved in the pathology associated with CoA deficiency.

## Discussion

CoA reduction is a pivotal event in PKAN and CoPAN patients, playing a critical role in the maintenance of mitochondrial function; however, the underlying mechanism remains elusive [22, 27–29]. The reduction of CoASY has been proposed as a potential cause for the mitochondrial impairment [28]. Impaired mitochondria can evidently disrupt ATP production, thereby compromising the energy supply, particularly in high-energy-demanding organs such as the muscle and brain [30]. Indeed, our study demonstrates that knockdown of Dpck renders flies more susceptible to energy shortage conditions, such as starvation. Interestingly, supporting evidence from ketogenic diet-treated Pank2^−/−^ mice further emphasizes the crucial role of energy shortage in inducing CoA deficiency-related neuropathology [31]. Furthermore, in addition to disrupting mitochondrial function, we propose that the downregulation of Dpck may impair mitochondrial functional integrity by compromising the integrity and activity of ETC complexes and reducing mitochondrial membrane potential, ultimately leading to a decrease in ATP production. The resulting energy deficiency can further contribute to the degenerative phenotype observed in both the muscle and nervous system due to their high energy demands, accompanied by an increase in apoptosis [21, 32].

It is intriguing to find that a deficiency in CoA can disrupt the integrity of ETC complexes, particularly complex I and III. Considering the involvement of CoA in regulating membrane modeling and gene expression [1, 14], it is plausible that disturbance of ETC integrity may be attributed to alterations in mitochondrial membrane composition and reduced expression levels of ETC subunits. However, further investigation is required to elucidate the underlying mechanism. Notably, we have collected some evidence at this stage, including the observed increase in mitochondrial DNA release and the decreased protein levels of certain ETC subunits in Dpck RNAi flies. Furthermore, CoA deficiency can disrupt lipid metabolism, particularly affecting membrane-associated lipid species [14]. Therefore, the reduction in ETC assembly and mitochondrial function may also be attributed to altered lipid species, such as a reduced synthesis of lipids and cholesterol [33]. Interestingly, some membrane lipid species, such as phosphatidylcholine (PC) and phosphatidylethanolamine (PE), which play a crucial role in the formation of the mitochondrial outer membrane, have been proposed to exert regulatory control over the activity of CoASY [34].

Although the mitochondrial abnormality is not observed in the muscle tissue of CoPAN patients, previous studies have reported the presence of giant mitochondria in the muscle tissue of PKAN patients [35]. Consistent with the findings in PKAN patients, our fly model exhibits aggregated mitochondria, particularly in muscles with Dpck RNAi, indicating that this mitochondrial aggregation serves as a hallmark reflecting impaired mitochondrial function resulting from CoA deficiency.

The increment of ROS has been demonstrated in both PANK and CoASY deficient cells and animals, indicating the potential pivotal role of redox species in the pathology associated with PANK and CoASY deficiencies [36–38]. Our findings also suggest that the reduction in CoASY levels leads to an increase in ROS sensitivity; however, the underlying mechanism remains unclear. Interestingly, oxidative stress can induce protein CoAlation, which serves as a protective mechanism against cysteine residue oxidation and is influenced by the level of CoA [39]. Consequently, the decrease in CoA levels may lead to a reduction in protective Cysteine-CoAlation, thereby increasing susceptibility to oxidative stress caused by elevated protein oxidation.

While both PANK2 and CoASY mutations result in iron accumulation in the human brain, PANK2 deficiency does not lead to iron accumulation in *Drosophila* [38]. Additionally, Dpck RNAi does not typically alter the expression of genes involved in regulating iron homeostasis (Supplementary Fig. 5), suggesting that iron accumulation does not occur in flies. Interestingly, we also employed TH-Gal4 and Gmr-Gal4 to selectively downregulate Dpck in dopaminergic neurons and retinal neurons within specific brain regions (Supplementary Fig. 6). Despite observing neurodegeneration upon Dpck RNAi, it is noteworthy that iron distribution and content likely remained unaltered, as demonstrated by the DAB-enhanced Perl’s staining and unchanged expression levels of Fer1, Fer2, and Mvl (Supplementary Fig. 6). Notably, considering the disparity in iron metabolism between *Drosophila* and humans, particularly the absence of key iron metabolism-related genes such as hepcidin, ferroportin, and transferrin receptor in flies, this phenomenon appears to be reasonable. Moreover, the brain iron accumulation phenotype also exhibits significant heterogeneity in individuals harboring the Dpck mutation, which has been proposed as a distinctive phenotype [32, 40].In addition, it also suggests that iron accumulation in PKAN and CoPAN patients may be attributed to the dysregulation of iron homeostasis-related genes that are not present in the fly genome. In fact, increased expression of transferrin receptor 1 and ferroportin has been observed in Hela cells with PANK2 RNAi [41], and patient iPSC-derived neuronal cells [42], providing further support for this possibility.

Iron accumulation in the globus pallidus is likely a secondary symptom, as evident by the absence of apparent neurological pathology in PANK2 null mice, despite detectable iron accumulation in their globus pallidus [17]. Moreover, the iron chelating agent deferiprone (3-hydroxy-1,2-dimethylpyridin-4-one, DFP) fails to alleviate the symptoms of PKAN [43], suggesting that iron accumulation may not significantly contribute to the neuropathology of NBIA.

Interestingly, long-term knockdown of Dpck in breast cancer cell lines does not exert any discernible impact on cellular proliferation and migration, which suggests that cancer cells possess the capability to sustain optimal levels of CoA [44]. Although we do not analyze cell proliferation in fly models, the ubiquitous knockdown of Dpck by Da-Gal4 indeed leads to developmental delay, suggesting that CoA plays a vital role in the developmental process. Several studies have reported that CoASY deficiency can impair development, not only in animal models [28], but also in human patients [40, 45]. Therefore, cancer cells may employ different strategies to overcome CoASY deficiency.

Currently, there is a lack of effective therapeutic interventions for the treatment of CoPAN and NBIA; however, several potential approaches have been proposed, such as the utilization of the iron chelator DFP and the drug pantethine to circumvent PANK2 deficiency [14, 46]. Considering that defective mitochondria are a common feature among several NBIA models [27], it is worth considering improving mitochondrial function as a potential strategy for ameliorating NBIA symptoms in the future. In particular, the simple compound dichloroacetic acid (DCA) and its derivatives, which have been utilized for the correction of inherited mitochondrial disorders [47], merit further consideration for inclusion in CoPAN and PKAN treatment.

## Materials and methods

### Fly stocks and genetics

Fly stocks and crosses were raised on the standard corn medium at 25 °C under ~ 65% humidity condition. UAS-Dpck RNAi flies (line 1#: THU3785. N, line 2#: B#35376) were obtained from the Tsinghua Fly Stock Center and Bloomington *Drosophila* Stock Centre (BDSC), respectively. Elav-Gal4 and MHC-Gal4 flies were acquired from BDSC, while Da-Gal4 was generously provided by Prof. Bing Zhou (Shenzhen Institute of Synthetic Biology, Shenzhen Institutes of Advanced Technology, Chinese Academy of Sciences). UAS-Dpck transgenes and UAS-hCoASY transgenes were generated through the injection of pUAST-Dpck-3×HA plasmids and pUAST-hCoASY-3×HA into *w*^*1118*^ embryos. To achieve ubiquitous knockdown and overexpression of fly Dpck gene, Da-Gal4 was crossed with UAS-Dpck RNAi and UAS-Dpck-3×HA, while Elav-Gal4 and MHC-Gal4 were crossed with UAS-Dpck RNAi and UAS-Dpck-3×HA to specifically target knockdown and overexpression in the CNS and indirect flight muscle. Corresponding controls were generated by crossing WT flies with Da-Gal4, Elav-Gal4, and MHC-Gal4, respectively.

### Pupation and eclosion rate measurement

To assess the pupation rate and eclosion rate, approximately 50 female and 40 male flies were transferred onto grape juice-agar plates for a 12-hour egg-laying period. Subsequently, 50 first instar larvae were collected and placed on standard corn medium with six replicates per genotype. The total number of pupae and adult flies was independently determined. The pupation rate was calculated as the ratio of the total number of pupae to the initial number of first instar larvae in each group, while the eclosion rate was obtained by dividing the total number of adult flies by the initial number of larvae.

### Lifespan recording, climbing ability, and behavior assay

To record the lifespan of flies, newly eclosed flies were collected and cultured at 29 ℃. For each genotype, a total of 100 flies were used, with fresh food being regularly replenished every three days. The number of dead flies was counted to determine the fly survival rate (%).

For the climbing ability assay, male WT, Dpck RNAi, and Dpck OE flies were divided into five groups, with 20 flies in each group. After being aged at 29 ℃ for four weeks, the fly climbing ability (%) was assessed by measuring the proportion of flies capable of climbing a distance of 6 cm within 5 s.

For the open field assay, a single male fly was placed on a glass disc with a diameter of 3.5 cm, and its movement trajectory was monitored for 10 min using the Labmaze Fly Trajectory Tracking System (ZHONGSHI SCIENCE & TECHNOLOGY). At least six individual flies were monitored per genotype, and the tracking system software (ZHONGSHI SCIENCE & TECHNOLOGY) was utilized to calculate the traveled distance and average speed.

### CoA content measurement

The CoA content was measured using the Coenzyme A Content Assay Kit (Abcam) following the manufacturer’s instructions. Briefly, 15–20 male flies, 20 fly heads, and thoraxes were collected, which were then homogenized in 200 µl CoA assay buffer on ice. Subsequently, centrifugation at 12,000 rpm for 10 min was performed to obtain the supernatant. Protein concentration was determined using BCA kits (Thermo Scientific).

Each well of a 96-well plate was filled with 60 µl of CoA assay buffer, followed by the addition of 30 µl supernatant. Subsequently, 2.5 µl of OxiRed probe was introduced to the reaction mixture and incubated in darkness for 30 min. The signal at 490/525nm was measured by EnVision Multilabel Plate Readers (Perkin Elme). At least three independent replicates were performed for each genotype.

### Annexin V-FITC and propidium iodide staining

Fly muscle and brain tissues were collected and dissected in 1xPBS (Sangon Biotech). Subsequently, the tissues were stained using the Annexin V-FITC/7-AAD Apoptosis Detection Kit (Beyotime Biotechnology) and Propidium iodide (Beyotime Biotechnology). The staining solution was prepared by combining 200 µl of Annexin V Binding Buffer, 5 µl of Annexin V-FITC, and 10 µl of Propidium iodide. The dissected tissues were incubated with the dyes at 37 °C for 1 h, followed by three washes with 1xPBS. Finally, visualization was performed using a ZEISS LSM 900 with Airyscan 2 confocal microscope (Zeiss).

### Immunofluorescence

The fly muscles and brains were collected and dissected in 1xPBS buffer, fixed with 4% paraformaldehyde (Absin), permeabilized with 0.3% Triton-X 100 (Sangon Biotech) in 1xPBS for 30 min, blocked with 10% normal goat serum (Absin) in 1xPBST (0.1% Tween20, Sangon Biotech) for one hour, and subsequently incubated overnight at 4 °C with the primary antibody (1:800 dilution).

The tissues were then washed three times with 1xPBST, followed by incubation with FITC-conjugated and TRITC-conjugated secondary antibodies against mouse and rabbit (Cell Signaling) at room temperature for 2 h. After three additional washes with 1xPBST, the stained tissues were mounted and visualized using the ZEISS LSM 900 with Airyscan 2 confocal microscope. The primary antibodies used were anti-ATP5a antibody (Abcam) and anti-TH antibody (Abcam).

### Brain section and H&E staining

After being incubated at 29 °C for 4 weeks, fly heads and thoraxes were collected and fixed overnight at 4 °C using carnoy fixation solution (ethanol: chloroform: acetic acid = 6:3:1, Sangon Biotech). Subsequently, they were dehydrated with anhydrous ethanol (Sangon Biotech), soaked in methyl benzoate (Sangon Biotech) overnight, and finally embedded in molten paraffin (Sinopharm Chemical Reagent). The embedded tissues were then sectioned into continuous sections of 8 μm using the Leica section apparatus (RM2235, Germany). To visualize the degenerative phenotype of tissues, hematoxylin and eosin (H&E) (ZSGB-BIO, China) staining was employed.

### TMRE staining

The TMRE staining solution was prepared by using the Mitochondrial Membrane Potential Assay Kit with TMRE (Beyotime Biotechnology) by diluting TMRE at a ratio of 1:1000 in detection buffer. The unfixed fly muscles and brains were collected and stained at room temperature in the dark for 30 min. After washing three times with 1xPBS, TMRE fluorescence was visualized by ZEISS LSM 900 with Airyscan 2 confocal microscope.

### Acetyl-CoA content assay

The levels of acetyl-CoA were quantified using an enzyme-linked immunosorbent assay (ELISA) kit (Sangon Biotech). Briefly, fly tissues were harvested and homogenized in 1×PBS buffer, followed by centrifugation to collect the supernatant. Protein concentration was determined using BCA kits (Thermo Scientific). Subsequently, a 100 µl sample was dispensed into a pre-coated 96-well plate with anti-acetyl-CoA antibody (Sangon Biotech) and incubated at 37°C for 90 minutes. After removing the liquid, a solution of biotinylated anti-acetyl-CoA antibody was added and incubated at 37°C for 60 minutes, followed by gentle washing four times. Then, 100 µl of horseradish peroxidase (HRP)-labeled streptavidin (Sangon Biotech) was added and incubated for 30 minutes before being washed four times again. Finally, 90 µl of the 3,3’,5,5’-tetramethylbenzidine (TMB) solution (Sangon Biotech) was added and allowed to react for 15 min; subsequently, the absorbance at 450 nm was measured.

### NADH and NAD+ content assay

The homogenization of 20 flies was performed in NAD+/NADH extraction buffer (Beyotime), followed by centrifugation to collect the supernatant. To convert NAD + to NADH, a mixture of 90 µl ethanol dehydrogenase working solution (Beyotime) and 20 µl sample was added to a 96-well plate and incubated at 37 °C for 10 min. Subsequently, 10 µl colorimetric solution (Beyotime) was added and incubated at 37 °C for an additional 30 min, after which the absorbance at 450 nm was measured to determine the total NAD content. Conversely, a portion of the sample (100 µl) underwent heat treatment in a water bath set at 60 °C for half an hour to remove NAD+, and its absorbance at 450 nm was measured accordingly. The relative contents of both NAD + and NADH were quantified.

### Triacylglycerol (TAG) content assay

Triacylglycerol (TAG) levels in flies were quantified using the TAG Content Assay Kit according to the manufacturer’s instructions (Sangon Biotech). Briefly, flies were collected and homogenized, followed by centrifugation to obtain the supernatant. Subsequently, sequential addition of TAG assay solutions was performed. The mixture was shaken for 30 s and repeated three times. After phase separation occurred at room temperature, 75 µl of the supernatant was transferred to a new tube. Assay solution III and solution IV were added, thoroughly mixed, and incubated in a water bath at 65 °C for 3 min. Following cooling, solution V and solution VI were added, mixed again, and incubated at 65 °C for 15 min. The absorbance at 420 nm was measured.

### Detection of free fatty acids (FFA)

The Free Fatty Acids (FFA) content was determined using the FFA Content Assay Kit (Solarbio LIFE SCIENCE). Twenty flies were homogenized in an extraction solution, followed by centrifugation to obtain the supernatant. Protein concentration was measured using a BCA assay kit (Thermo Scientific). Solution I, composed of n-hexane, anhydrous methanol, and chloroform in a 24:21:25 ratio, along with solution II and 30 µl of the supernatant were added and mixed. The mixture was incubated at 37 °C for 15 min and then centrifuged at 3,000 rpm for 10 min. The upper phase was transferred and combined with solution III. After shaking for 5 min, the mixture was transferred to a 96-well plate and the absorption at 550 nm was measured to determine the FFA content.

### Muscle lipid droplets (LDs) staining

The fly muscles were dissected in 1×PBS (Sangon Biotech) and fixed in 4% paraformaldehyde (Absin). Subsequently, they were permeabilized for 30 min in 1×PBS containing 0.3% Triton-X 100 (Sangon Biotech), followed by staining with a solution of 0.1% BODIPY 493/503 (Beyotime) in 1× PBS for a duration of 20 min. After being washed three times, the muscle tissues were stained with a solution of 0.5% Actin Tracker Red Rhodamine (Beyotime) for a period of 30 min. Fluorescent images were acquired using ZEISS LSM900 with Airyscan2 confocal microscope at excitation wavelengths of 488 nm and 540 nm.

### 3,3’-diaminobenzidine (DAB) enhanced iron staining

The brains of Drosophila were dissected, and then fixed in 4% paraformaldehyde for 30 minutes, followed by three washes with 1×PBS. Subsequently, the samples were incubated at 37°C for 2 hours in a freshly prepared Prussian blue staining solution (20% HCl:10% K4Fe(CN)6, Sangon Biotech) to detect ferric iron. After three additional washes, the staining was intensified by incubating the samples in 1×PBS containing 0.025% 3,3’-diaminobenzidine (DAB) and 0.005% H2O2 for a duration of 20 min. Following another round of three washes with 1×PBS, iron distribution within different brain regions was visualized using an optical microscope and quantified through measurements of intensity.

### RT-PCR analysis

20 flies and corresponding tissues were collected for RNA extraction using the Trizol reagent (Invitrogen™). Equal amounts of RNAs from each genotype were reverse transcribed using the HiScript III All-in-One RT SuperMix Perfect for qPCR kit (Vazyme) in a 20 µl reaction system. The resulting cDNA was then used for RT-PCR experiments with Green Taq Mix (Vazyme), utilizing the primers listed below: *rp49*-F: 5-GCACCAAGCACTTCATCC-3, *rp49*-R: 5-CGATCTCGCCGCAGTAAA-3, *Dpck*-F: 5-CTGGACAAGAAGTGCGAGAAG-3, and *Dpck*-R: 5-GAAGCCCACGATTAGGAACA-3.

### Western blot and blue native PAGE (BN-PAGE) analysis

20 aged flies and tissues in each group were collected and homogenized in 150 µl protein lysis solution (Absin) on ice, containing the protease inhibitor (Absin). Subsequently, the samples were centrifuged at 4 ℃ for 10 min at 12,000 rpm. The resulting supernatants were mixed with 6xSDS-protein loading buffer (Transgen Biotech) and boiled for 10 min. 20–50 µg proteins were loaded onto the SDS-PAGE gel (Sangon Biotech), followed by transfer onto a PVDF membrane (Merck-Millipore). For immunoblotting analysis, primary antibodies against Tubulin (Abcam), ATP5a (Abcam), CI-30 (Abcam), C-IV s.1(Abcam), Cox5a(Abcam), and NDUFA1 (Santa Cruz Biotechnology) were used. Following overnight incubation with the primary antibodies at 4 ℃, the membranes were washed with 1xPBST before being further incubated with HRP-conjugated anti-mouse and anti-rabbit secondary antibodies (Cell Signaling). Finally, the HRP signal was detected using an Ultra High Sensitivity ECL Kit (MedChem Express).

For Blue native PAGE (BN-PAGE) analysis, at least 40 thoracic tissues were collected and subjected to protein extraction using lysis buffer (Absin). The extracted proteins were then mixed with 6x non-denaturing protein loading buffer (TransGen Biotech) before being loaded onto BIS-Tris Gradient Gels (4-20% concentration, Absin) for protein separation. Subsequently, the gels were stained with Coomassie Brilliant Blue (Sangon Biotech) for 2 h and subsequently washed three times using a destaining solution composed of 100 ml acetic acid (Sinopharm Chemical Reagent), 100 ml ethanol (Sinopharm Chemical Reagent), and dH_2_O adjusted to a final volume of 1 L.

### ATP content determination

To analyze the ATP content, 15 flies and 20 fly tissues were collected for each group and subjected to ATP quantification using an ATP content assay kit (Beyontime Biotechnology). Briefly, 200 µl lysis solution was added, followed by homogenization of the flies and tissues on ice. The resulting homogenate was then centrifuged at 4 ℃, 12,000 rpm for 5 min to collect the supernatant. Protein concentration was measured using a BCA kit. Subsequently, 100 µl of ATP assay buffer and 30 µl of supernatant were added to a 96-well plate. The NADPH content was immediately measured at 340 nm using EnVision Multilabel Plate Readers (Perkin Elmer) to determine the ATP levels.

### Detecting the activity of mitochondrial ETC complexes

For each group, 50 thoracic tissues from flies were collected and homogenized on ice within 500 µl mitochondria extraction buffer (250mM Scurose, 5mM EGTA, 10µM Tris-HCl, 10% BSA (Sangon Biotech) and 0.5% protease inhibitor (Absin)). After centrifugation at 4 ℃ and 1,000 rpm for 10 min to remove debris, mitochondria were isolated by further centrifugation at 4 ℃ and 10,000 rpm for 15 min. Protein concentration was quantified using the BCA kit. The activity of mitochondrial electron transport chain (ETC) complexes I-IV was assessed using the Mitochondrial Respiratory Chain Complex Activity Assay Kit (Solarbio LIFE SCIENCE) on a 96-well plate, following the manufacturer’s instructions. The decrease in optical density (OD) at 340 nm from NADH to NAD + was measured for Complex I, while for Complex II, the decrease in OD at 605 nm from Coenzyme Q to Reduced Coenzyme Q was determined. Additionally, the change in OD at 550 nm from reduced Cytochrome C to Oxidized Cytochrome C was quantified for Complex IV, and the increase in OD at 550 nm from Oxidized Cytochrome C to Reduced Cytochrome C was measured for Complex III using EnVision Multilabel Plate Readers (Perkin Elme). The activity of the mitochondrial ETC complex was calculated.

### Starvation and ROS treatment

To assess the starvation sensitivity of flies, a total of 50 female and 40 male flies were mated on grape juice plates with 2% agar. After laying eggs for 48 h, the parental flies were removed, and third instar larvae were carefully collected and transferred onto a medium containing solely 2% agar. Subsequently, the pupation rate and mortality were recorded, and the climbing ability was assessed after starving for 24 h.

In order to assess the sensitivity to reactive oxygen species (ROS), male flies aged 3–5 days were collected and transferred onto food containing 5 µg/ml tert-butyl hydrogen peroxide (TBHP, Sangon Biotech). The survival rate and climbing ability were recorded as previously described, with climbing ability measured after a 24-hour TBHP treatment.

### Statistical analysis

Statistical analyses were performed using GraphPad Prism (v9.0), and significance was determined by conducting a t-test analysis for two groups and ANOVA test for more than three groups. The data are presented as the mean ± S. E. M. The Gehan-Breslow-Wilcoxon test was employed to examine the disparity in survival rates (Figs. 1I and 7A and I).

## Electronic supplementary material

Below is the link to the electronic supplementary material.

Supplementary Material 1

## Data Availability

Data is available on request from the corresponding author.
