# Supplementary material for: Impaired mitochondrial integrity and compromised energy production underscore the mechanism underlying CoASY protein-associated neurodegeneration
Source: Cell Mol Life Sci. 2025 Feb 22;82(1):84. doi: 10.1007/s00018-025-05576-1 (PMC11846818; doi:10.1007/s00018-025-05576-1)
Supplement: Supplementary file 1 — Supplementary Material 1 [file 18_2025_5576_MOESM1_ESM.docx]

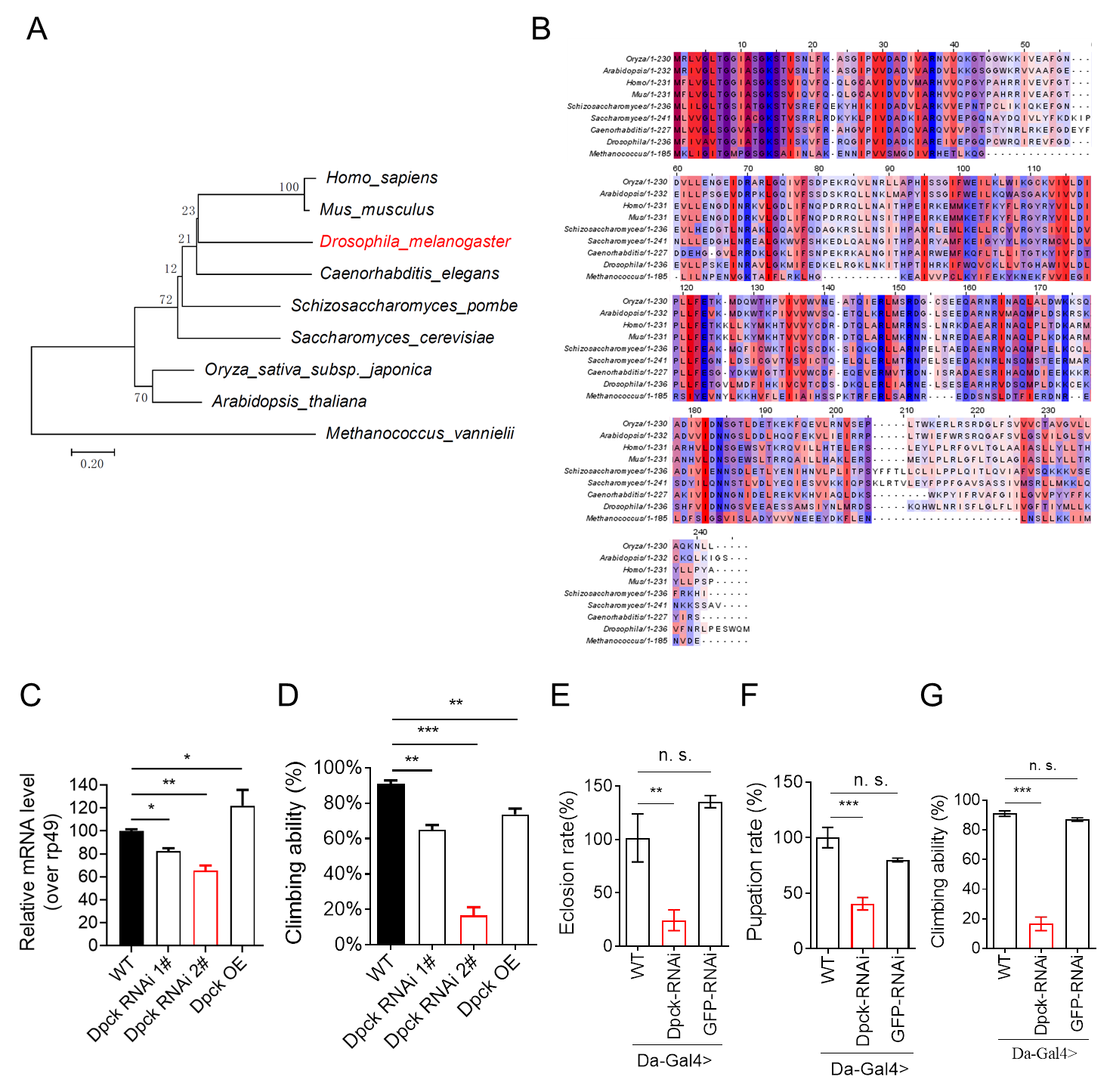


**Supplemetary Fig. 1. Dpck is evolutionarily conserved**

**(A).** Phylogenetic analysis of Dpck across different species. Distance bar = 0.2. The NJ tree is constructed using the MEGA7 software. **(B)**. Multiple sequence alignments of Dpck protein sequences. **(C)**. The efficiency of Dpck RNAi and overexpression is analyzed by RT-PCR. *: p < 0.05, **: p < 0.01. Da-Gal4 is used to drive the ubiquitous expression and knockdown of Dpck. **(D)**. The climbing ability of Dpck OE and RNAi flies. **: p < 0.01, ***: p < 0.001. Da-Gal4 is used to drive the ubiquitous expression and knockdown of fly Dpck. **(E-G)**. The eclosion rate, pupation rate, and climbing ability of UAS-GFP-RNAi flies. Da-Gal4 is used to drive the ubiquitous knockdown of GFP. **: p < 0.01; ***: p < 0.001. **(E)** eclosion rate, n = 250 (5 biological replicates). **(F)** pupation rate, n = 300 (6 biological replicates). **(G)** Climbing ability, n = 100 (5 biological replicates).


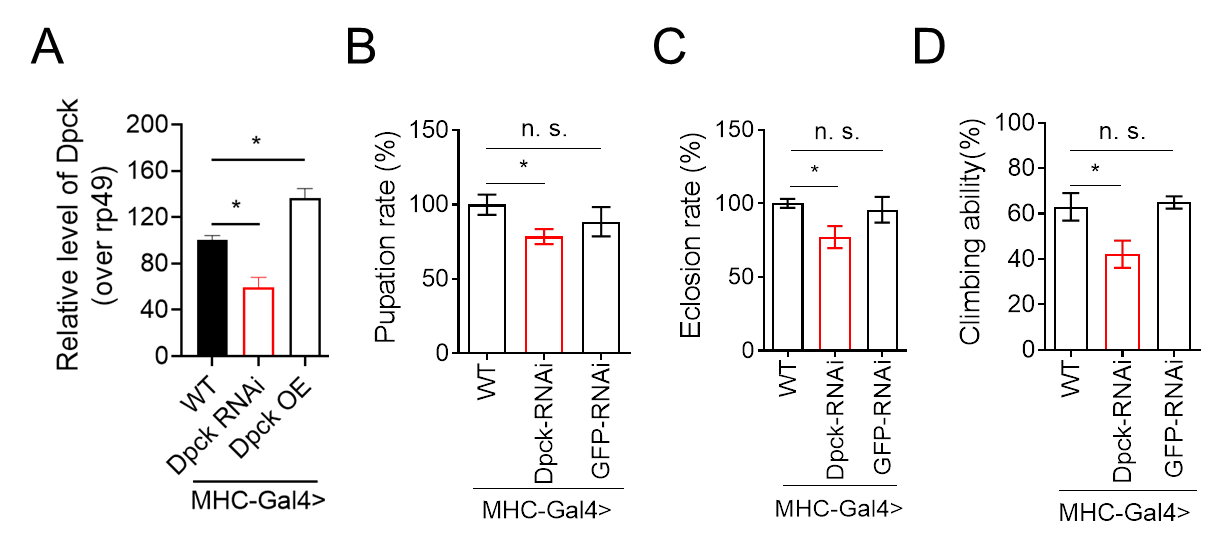


**Supplementary Fig. 2.** **The efficacy of tissue-specific Dpck knockdown. (A)**. The efficacy of muscle-specific OE and knockdown of Dpck is analyzed by RT-PCR. *: p < 0.05. MHC-Gal4 is used to drive the OE and knockdown of Dpck in fly indirect flight muscles. n = 20 (3 biological replicates). **(B-D)**. The eclosion rate, pupation rate, and climbing ability of UAS-GFP-RNAi flies. MHC-Gal4 was utilized to induce the knockdown of GFP specifically in the indirect flight muscles.*: p < 0.05. **(B)** pupation rate, n = 250 (5 biological replicates). **(C)** eclosion rate, n = 250 (5 biological replicates). **(D)** climbing ability, n = 100 (5 biological replicates).


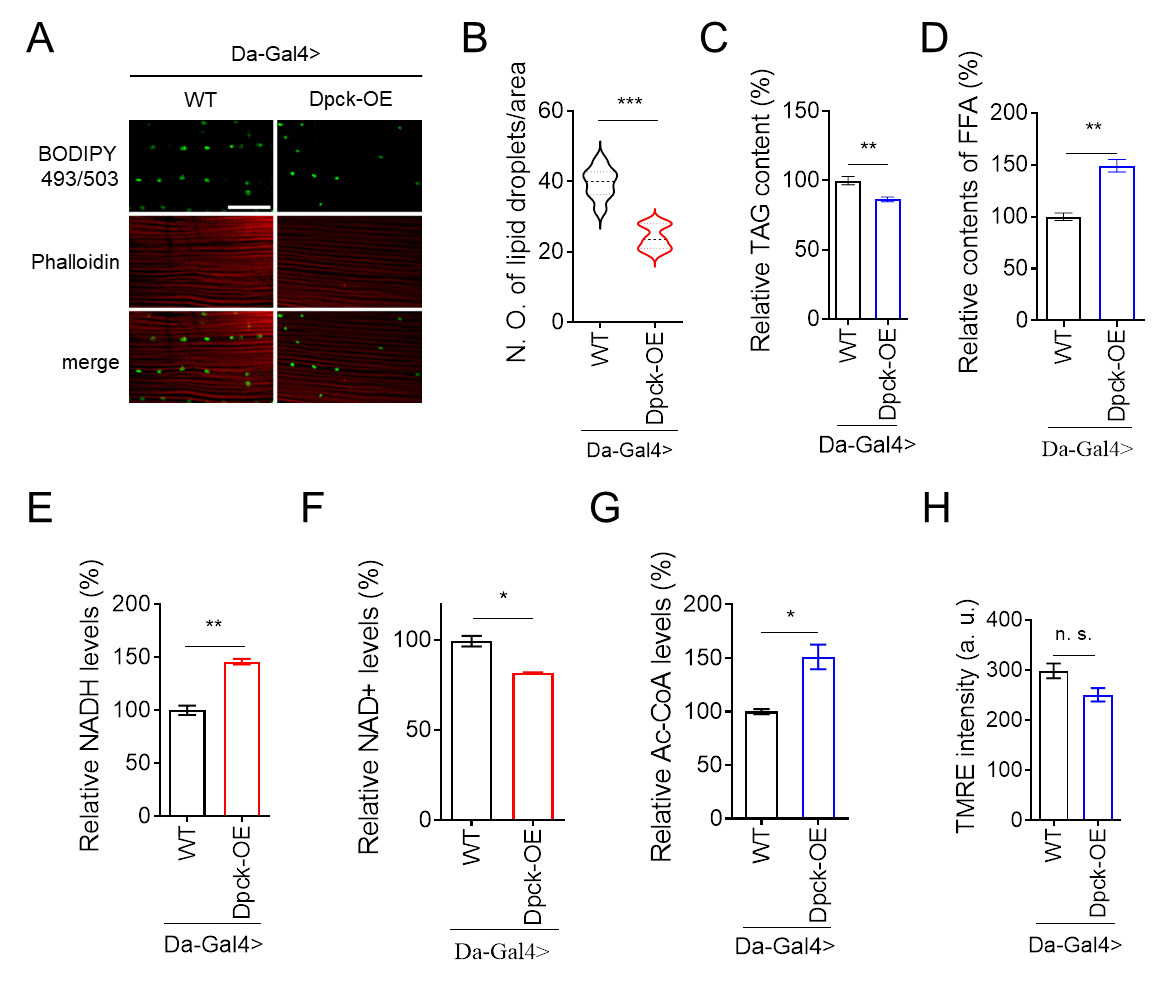


**Supplementary Fig. 3. Dpck OE improves lipid metabolism.**

**(A)**. The assessment of muscle lipid droplets (LDs) is conducted through staining with BODIPY 493/503. Da-Gal4 is employed to induce the overexpression of Dpck. *: p < 0.05. n = 8. Scale bar = 50um. **(B)**. is the quantitative results of **(A)**. ***: p < 0.001. **(C, D)**. The levels of triacylglycerol (TAG) and free fatty acid (FFA) are quantified in Dpck OE flies. **: p < 0.01. **(C)**. TAG content, n = 20 (3 biological replicates). **(D)**. FFA content, n = 20 (3 biological replicates)**. (E, F)**. The levels of NADH and NAD + in Dpck overexpressing flies are quantified. *: p < 0.05; **: p < 0.01. n = 20 (3 biological replicates). **(G)**. The levels of Acetyl-CoA in Dpck OE flies are measured. *: p < 0.05. n = 20 (3 biological replicates)**. (H)**. The mitochondrial membrane potential in Dpck overexpressing flies is assessed by quantifying the TMRE fluorescence intensity. The Da-Gal4 driver is employed to induce the overexpression of Dpck. n = 20 (3 biological replicates).


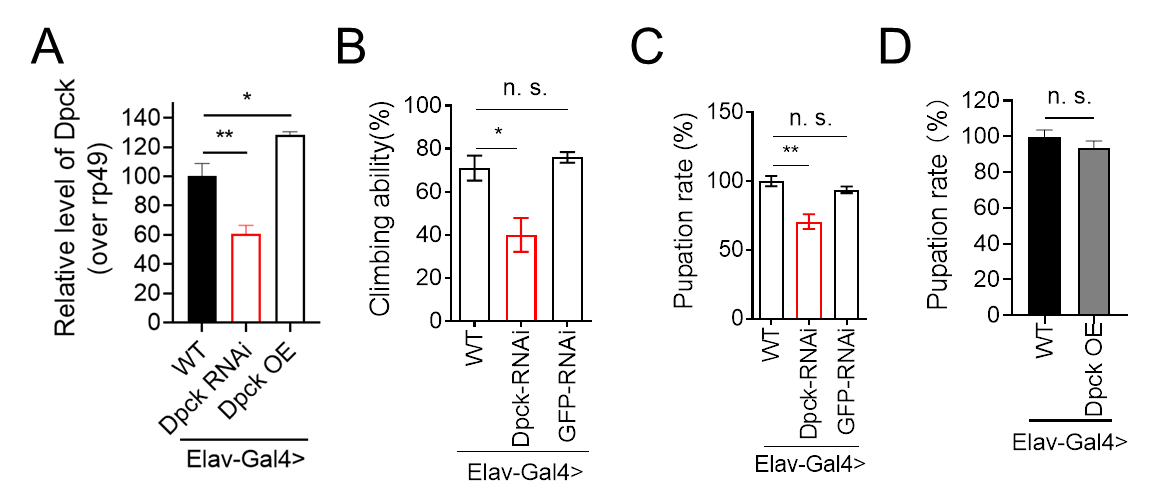


**Supplementary Fig. 4. The effectiveness of central nervous system (CNS)-specific overexpression and downregulation of Dpck..**

**(A)**. The efficacy of central nervous system (CNS)-specific OE and knockdown of Dpck is analyzed by RT-PCR. *: p < 0.05. Elav-Gal4 is used to drive the OE and knockdown of Dpck in the fly's central nervous system (CNS). **(B, C)**. The pupation rate and climbing ability of UAS-GFP-RNAi flies. Elav-Gal4 is employed to induce GFP knockdown in the CNS of the flies. *: p < 0.05; **p < 0.01. **(D)**. The pupation rate of flies with CNS-specific overexpression of Dpck. **: p < 0.01.

**Supplementary Fig. 5. Expression levels of iron metabolism-related genes.**

The expression levels of iron metabolism-related genes were analyzed by RT-PCR. *: p < 0.05. Da-Gal4 is used to drive the ubiquitous knockdown of Dpck. n = 20 (3 biological replicates).

**
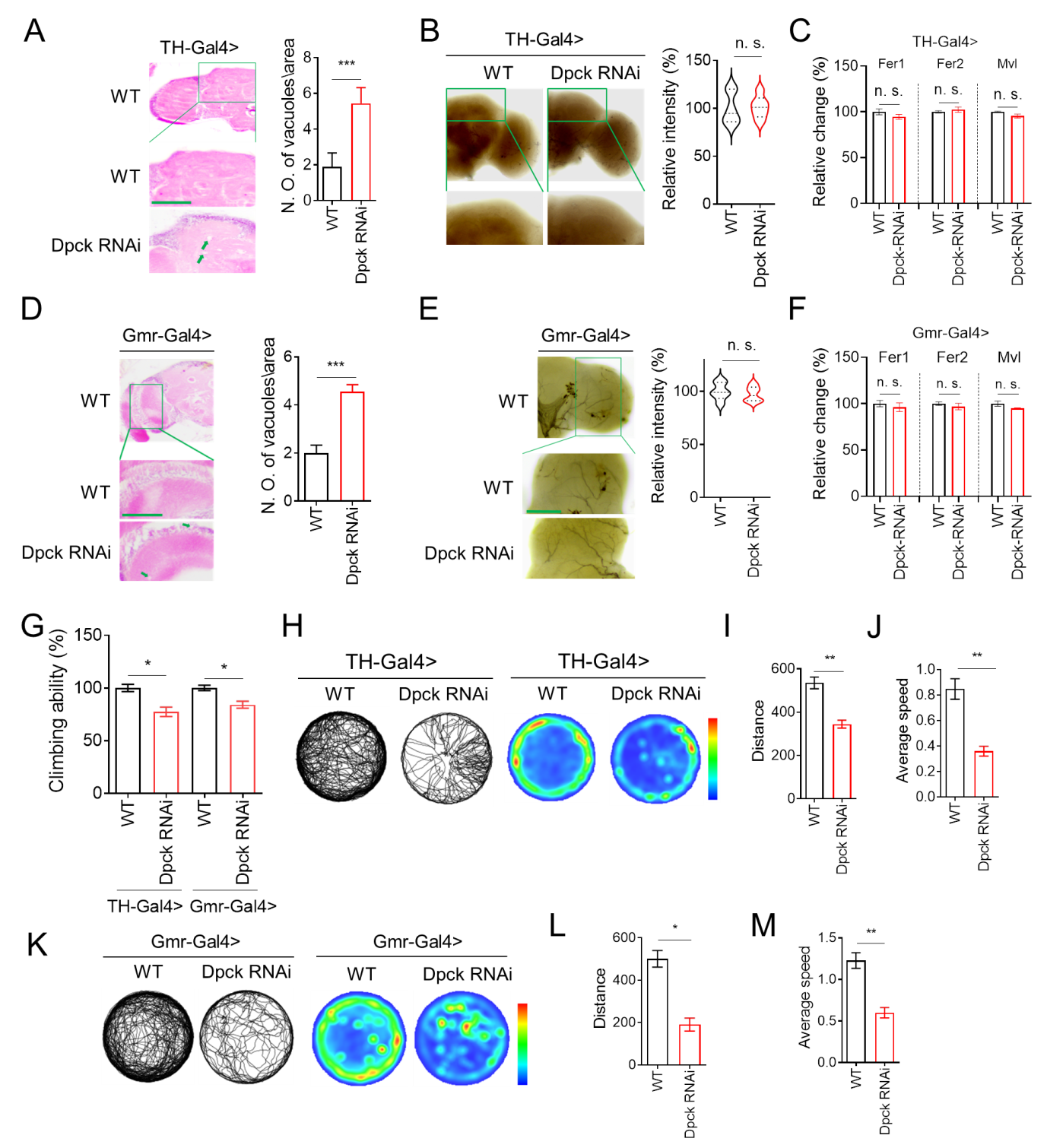
**

**Supplementary Fig. 6. Knockdown of Dpck in specific neurons does not alter iron homeostasis.**

**(A-C).** Dpck is specifically knocked down in dopaminergic neurons by using TH-Gal4. **(A).** H&E staining demonstrates degenerative phenotypes in the brain section of Dpck RNAi flies, indicated by arrows. Scale bar = 80 um. The number of degenerative vacuoles are counted and presented on right panel. ***: p < 0.001. n = 20. **(B).** DAB-enhanced Perl’s iron staining is used to analyze iron distribution in fly brain. Intensity of DAB signals is quantitated and presented on right panel. n = 4 (6 biological replicates). **(C)**. Expression levels of iron homeostasis-related genes are analyzed by qPCR. n = 20 (3 biological replicates). **(D-F)**. Dpck is specifically knocked down in retinal neurons by using the Gmr-Gal4. **(D)**. H&E staining demonstrates degenerative phenotypes in the medulla of Dpck RNAi flies, indicated by arrows. Scale bar = 80 um. The number of degenerative vacuoles are counted and presented on right panel. ***: p < 0.001. n = 20. **(E)**. DAB-enhanced Perl’s iron staining is used to analyze iron distribution in fly brain. Intensity of DAB signals is quantitated and presented on right panel. n = 4 (6 biological replicates). **(F)**. Expression levels of iron homeostasis-related genes are analyzed by qPCR. n = 20 (3 biological replicates). **(G)**. The climbing ability of flies with dopaminergic neuron-specific Dpck RNAi. *: p < 0.05. n = 100 (5 biological replicates). **(H-J)**. Open-field analysis of flies with dopaminergic neuron-specific Dpck RNAi. n = 4. **(I)**. moved distance and **(J)** average speed are the quantitative results of (H). **: p < 0.01. **(K)**. The climbing ability of flies with retinal neuron-specific Dpck RNAi. *: p < 0.05. **(L-N)**. Open-field analysis of flies with retinal neuron-specific Dpck RNAi. n = 4. **(M)**. moved distance and **(N)** average speed are the quantitative results of **(L)**. *: p < 0.05; **: p < 0.01.
